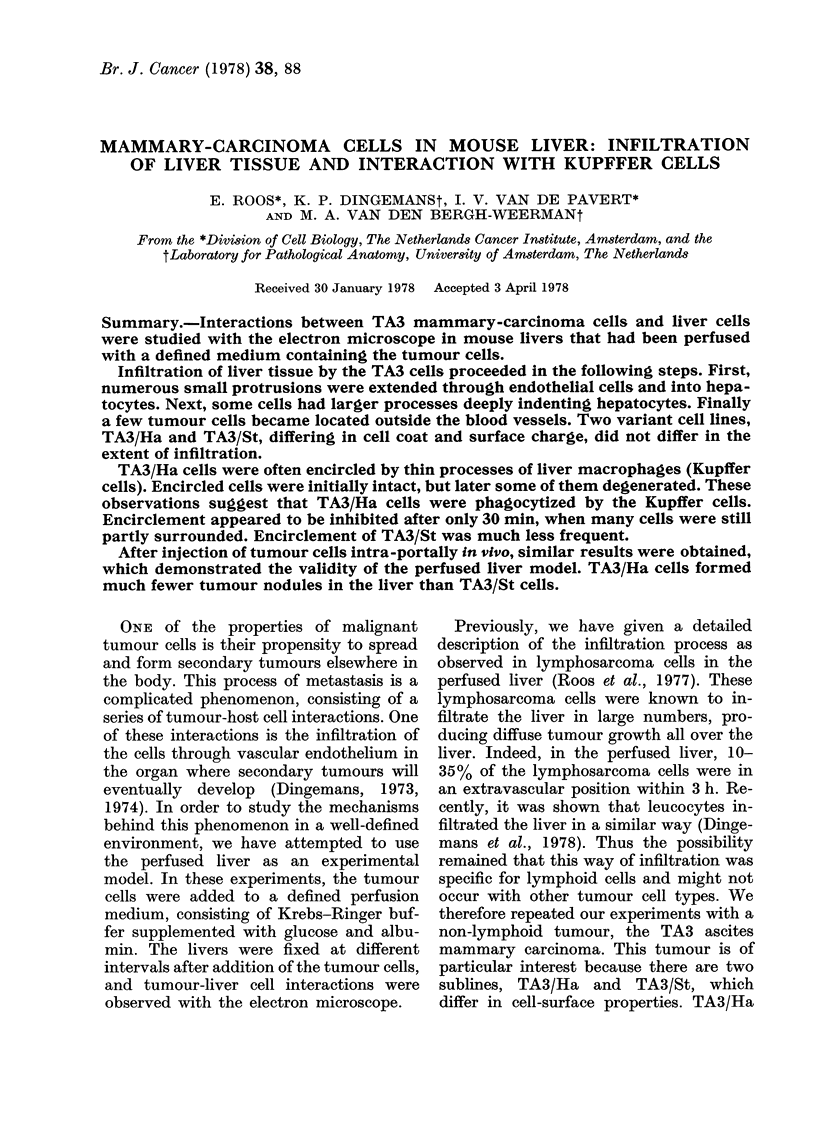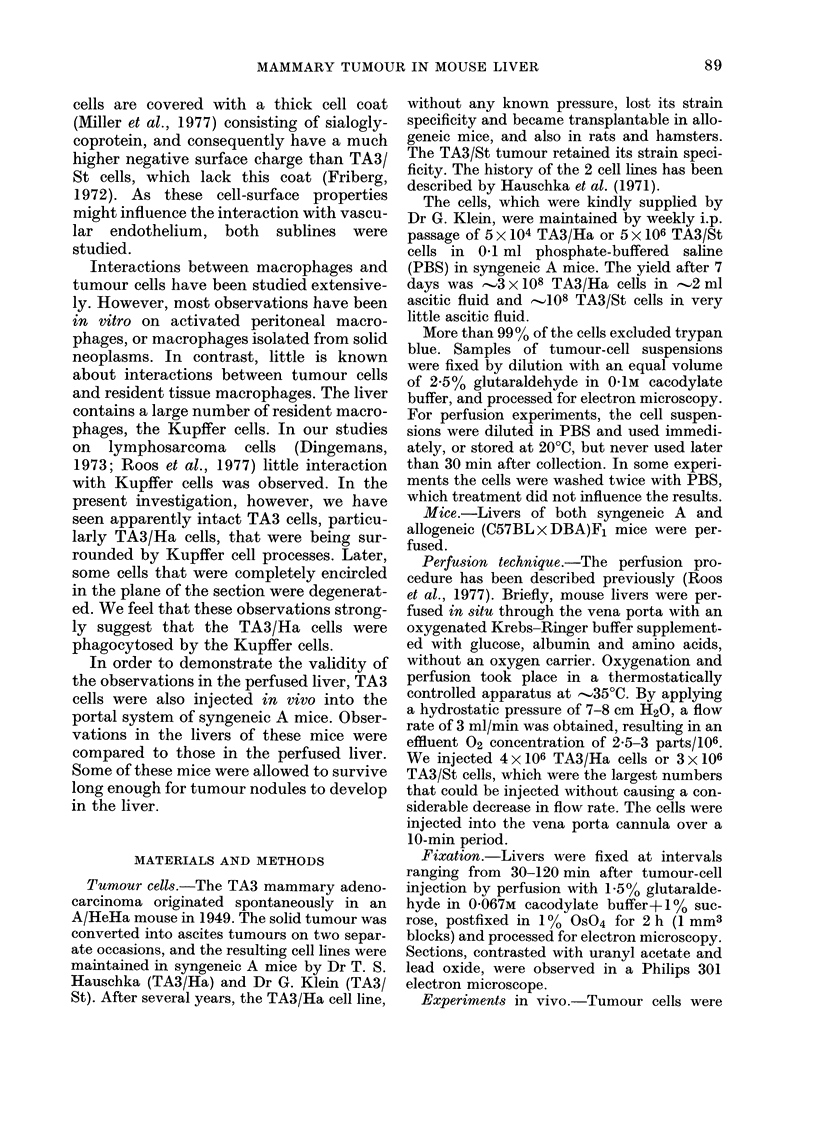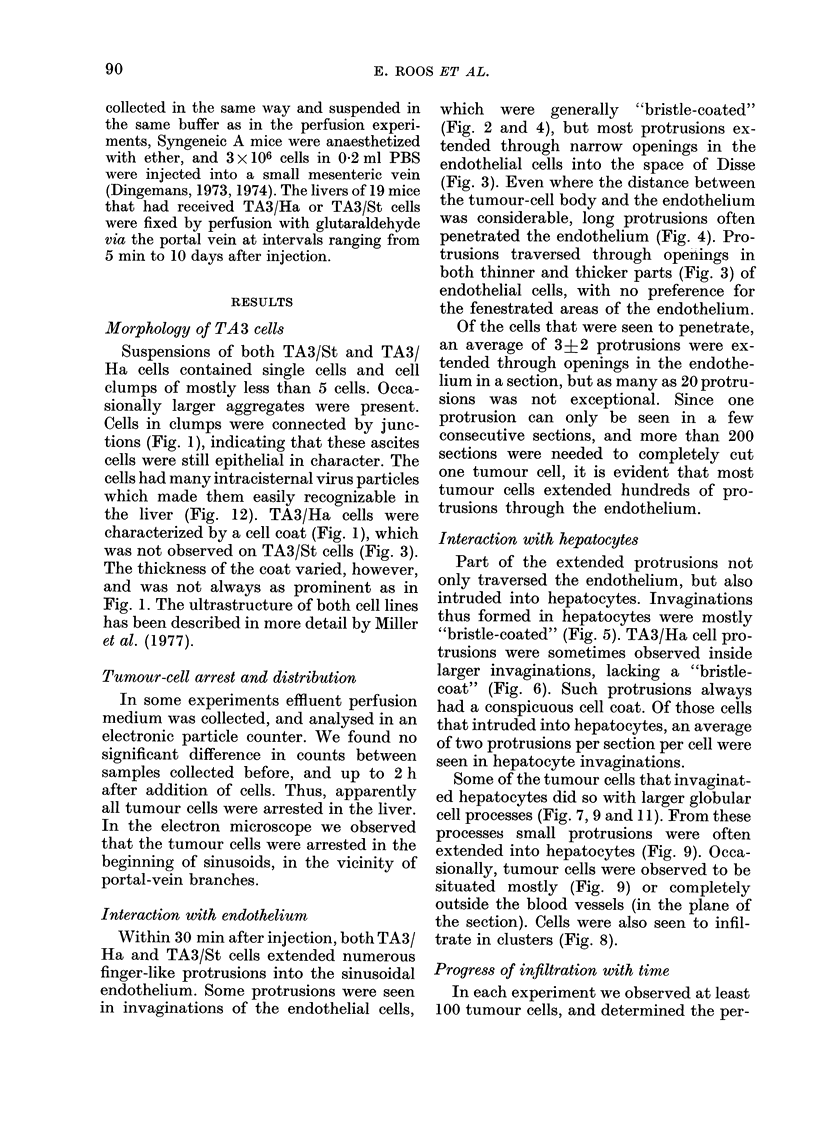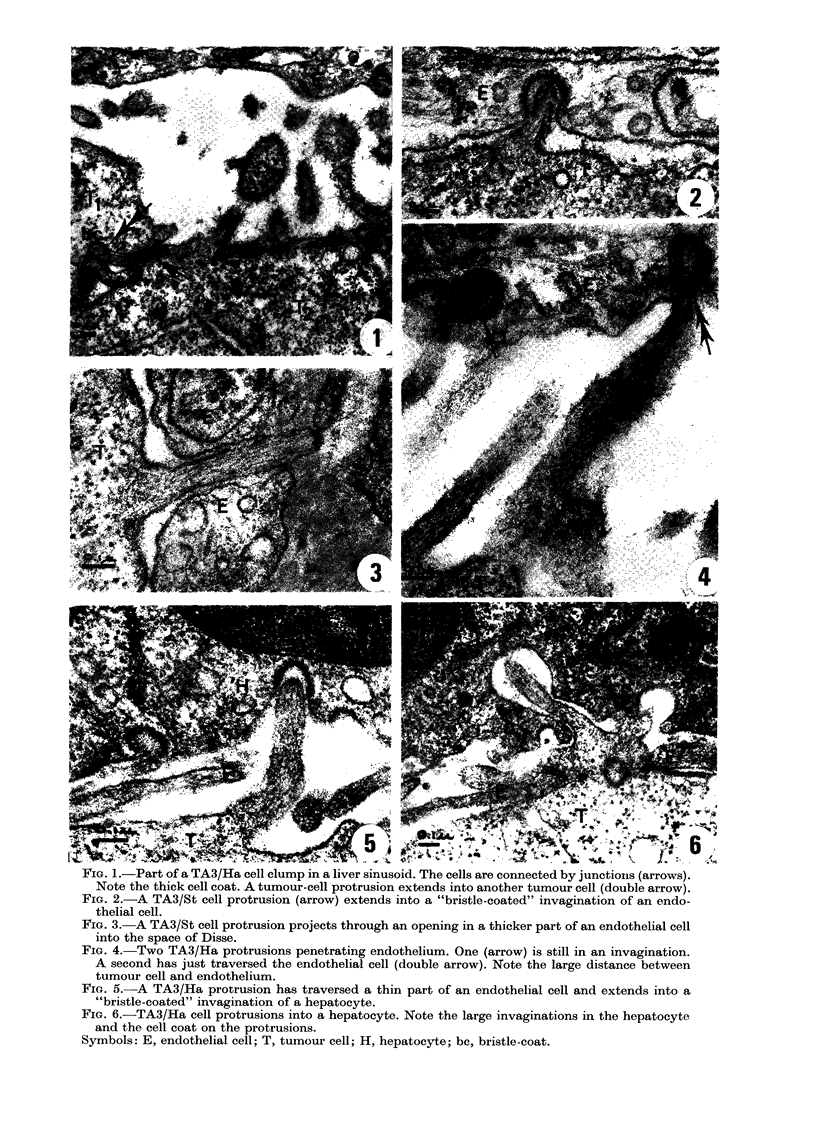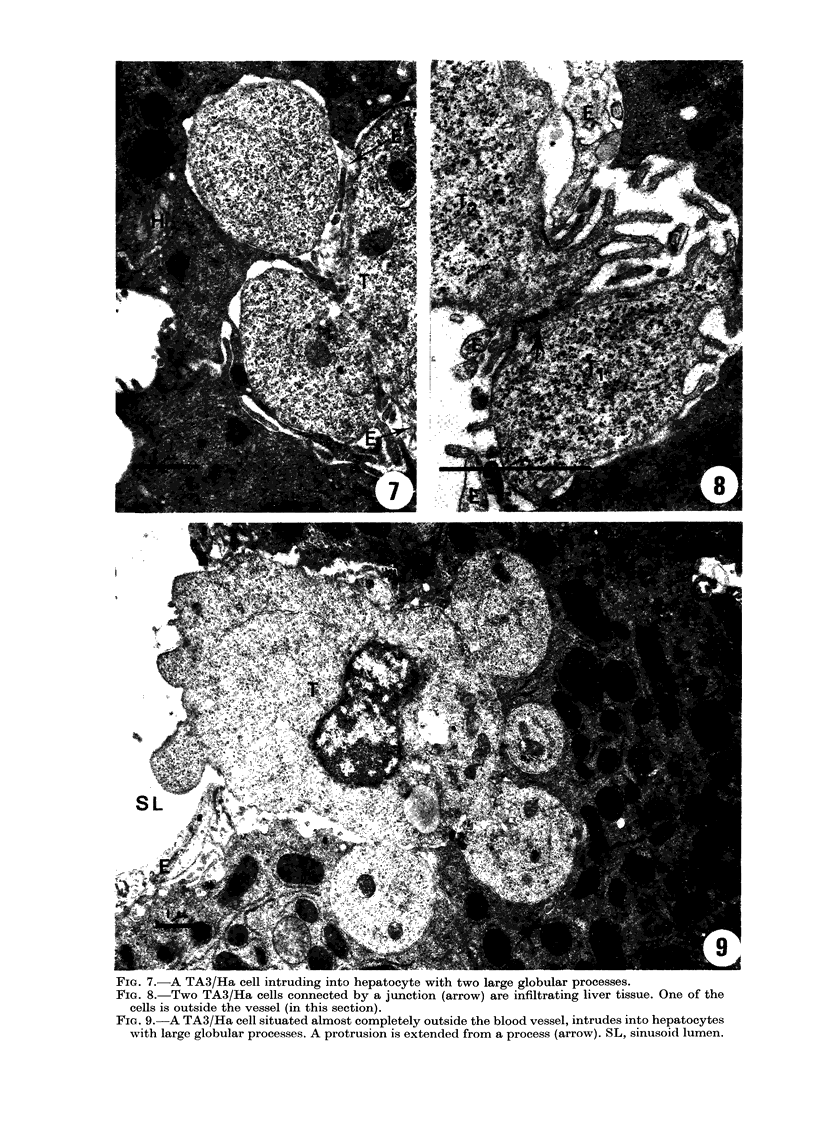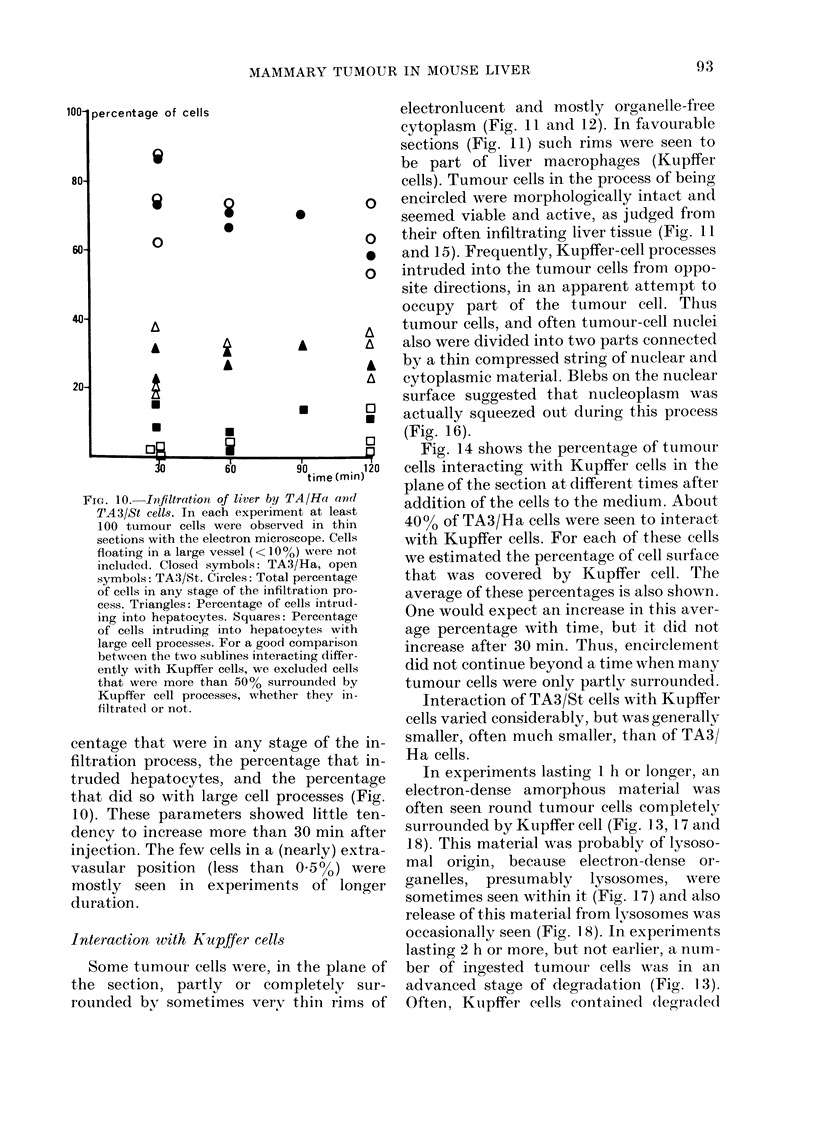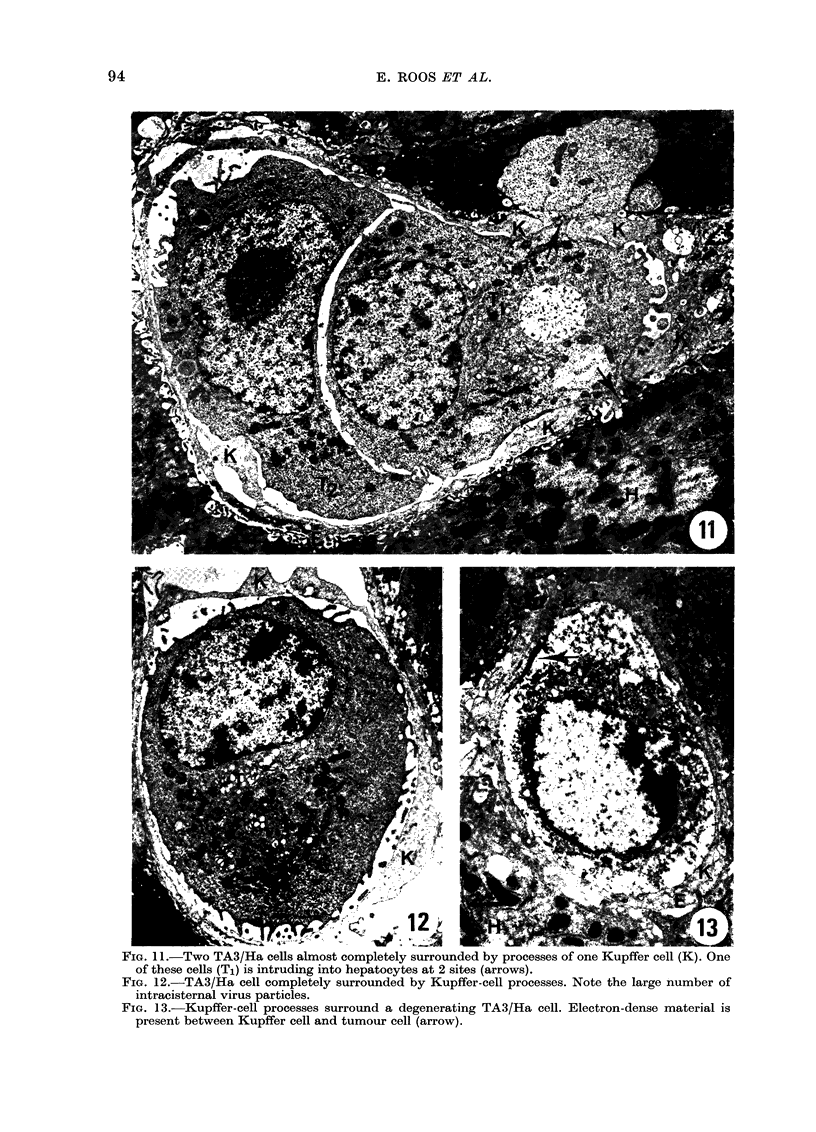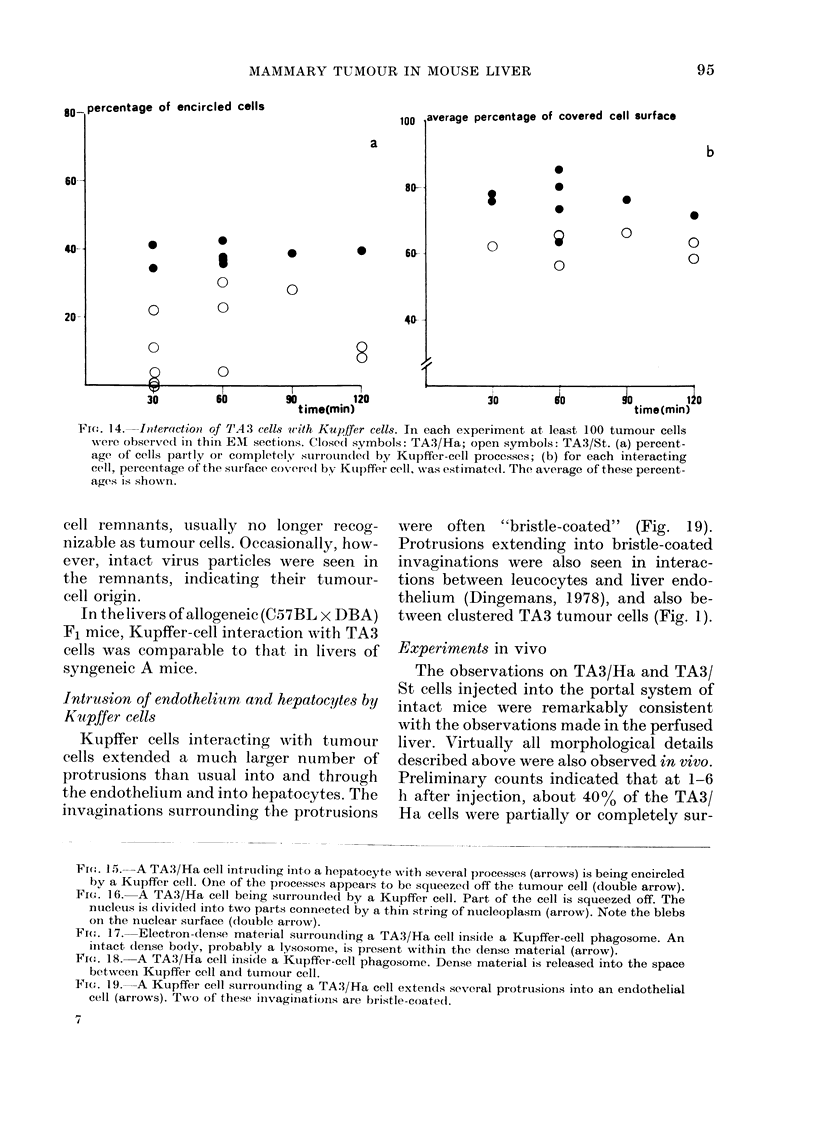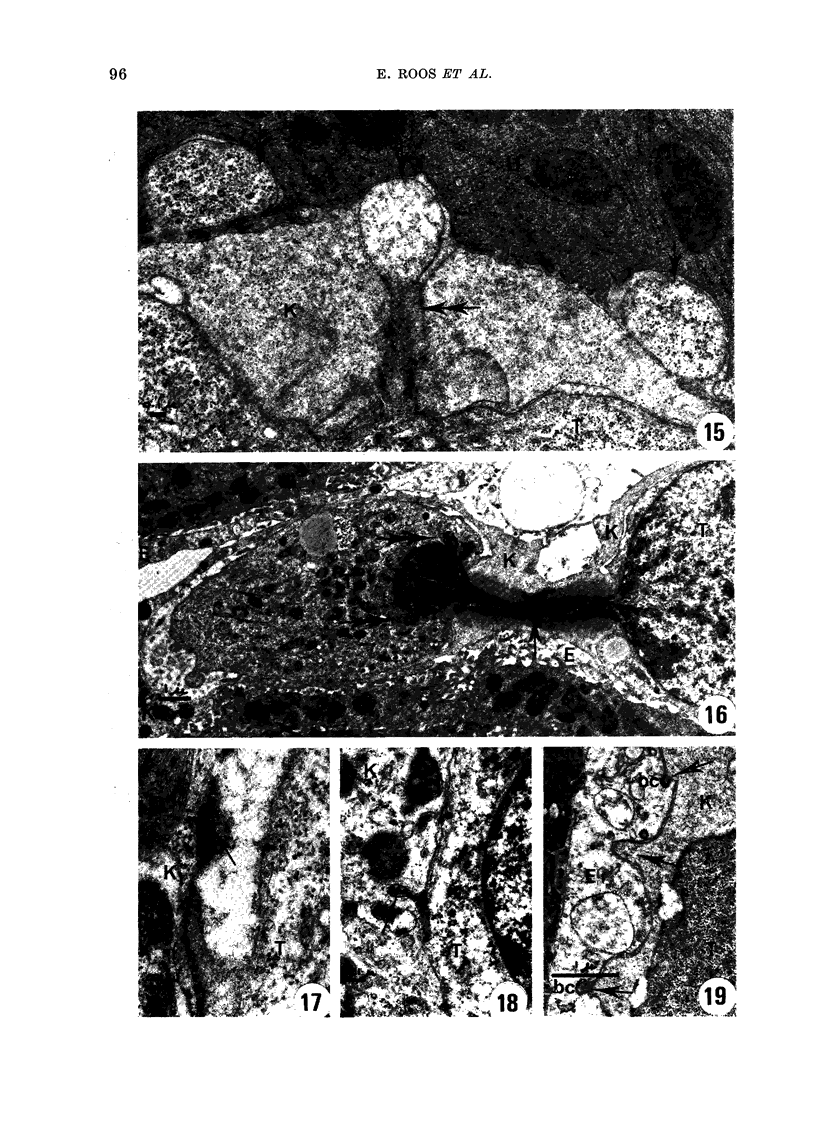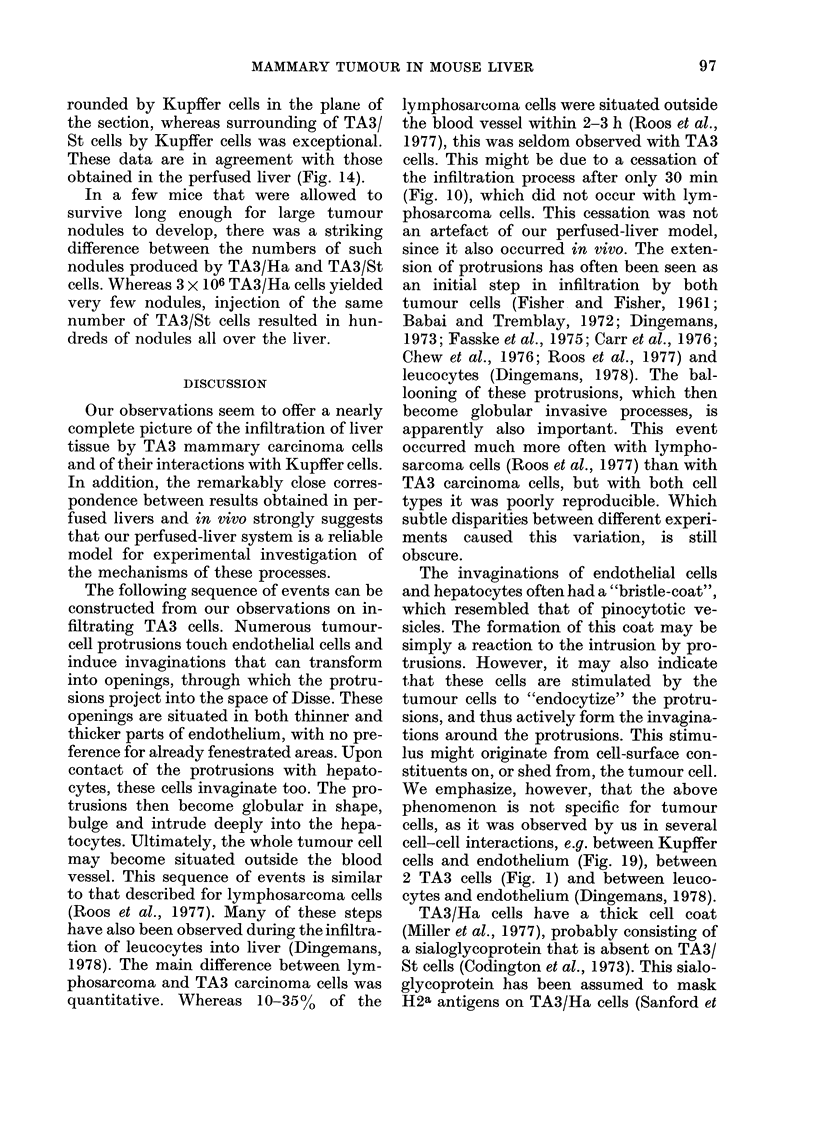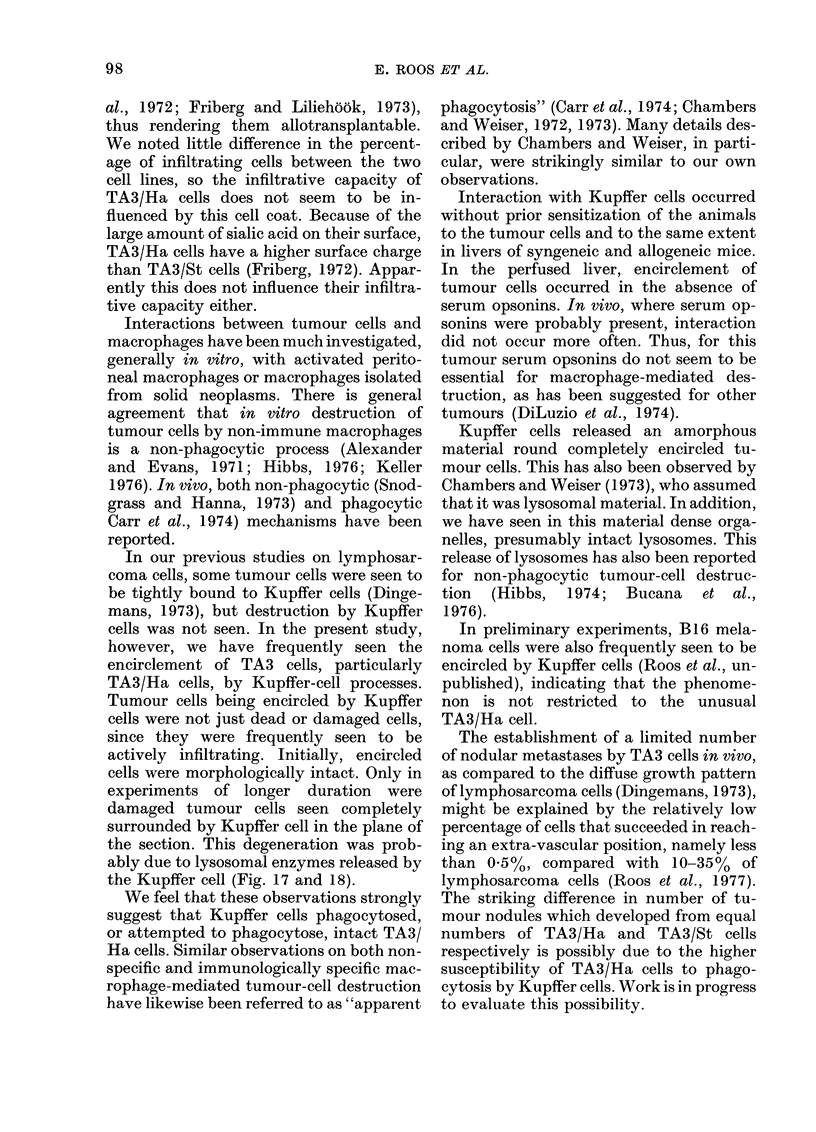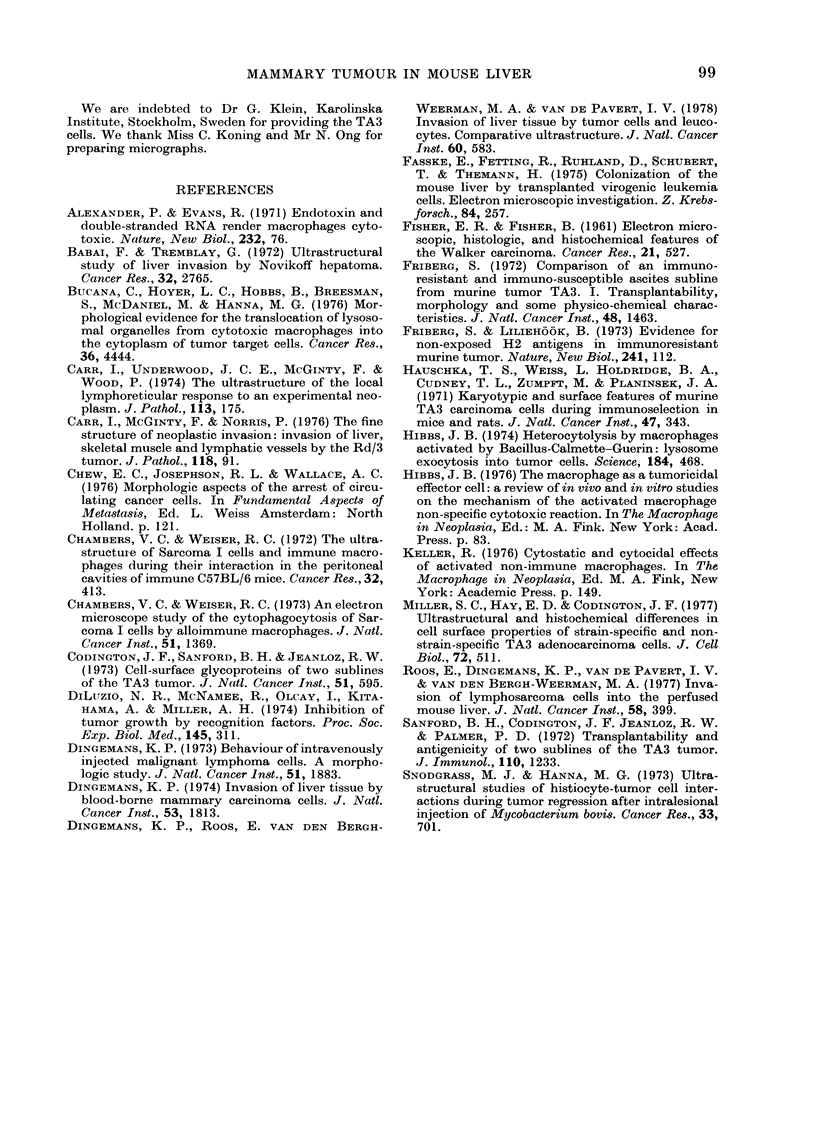# Mammary-carcinoma cells in mouse liver: infiltration of liver tissue and interaction with Kupffer cells.

**DOI:** 10.1038/bjc.1978.167

**Published:** 1978-07

**Authors:** E. Roos, K. P. Dingemans, I. V. Van de Pavert, M. A. Van den Bergh-Weerman

## Abstract

**Images:**


*Br. J. Cancer* (1978) **38**, 88

### MAMMARY-CARCINOMA CELLS IN MOUSE LIVER: INFILTRATION OF LIVER TISSUE AND INTERACTION WITH KUPFFER CELLS

E. ROOS*, K. P. DINGEMANS†, I. V. VAN DE PAVERT* AND M. A. VAN DEN BERGH-WEERMAN†

*From the *Division of Cell Biology, The Netherlands Cancer Institute, Amsterdam, and the †Laboratory for Pathological Anatomy, University of Amsterdam, The Netherlands*

Received 30 January 1978 Accepted 3 April 1978

**Summary.—Interactions between TA3 mammary-carcinoma cells and liver cells were studied with the electron microscope in mouse livers that had been perfused with a defined medium containing the tumour cells.**

**Infiltration of liver tissue by the TA3 cells proceeded in the following steps. First, numerous small protrusions were extended through endothelial cells and into hepatocytes. Next, some cells had larger processes deeply indenting hepatocytes. Finally a few tumour cells became located outside the blood vessels. Two variant cell lines, TA3/Ha and TA3/St, differing in cell coat and surface charge, did not differ in the extent of infiltration.**

**TA3/Ha cells were often encircled by thin processes of liver macrophages (Kupffer cells). Encircled cells were initially intact, but later some of them degenerated. These observations suggest that TA3/Ha cells were phagocytized by the Kupffer cells. Encirclement appeared to be inhibited after only 30 min, when many cells were still partly surrounded. Encirclement of TA3/St was much less frequent.**

**After injection of tumour cells intra-portally *in vivo*, similar results were obtained, which demonstrated the validity of the perfused liver model. TA3/Ha cells formed much fewer tumour nodules in the liver than TA3/St cells.**

One of the properties of malignant tumour cells is their propensity to spread and form secondary tumours elsewhere in the body. This process of metastasis is a complicated phenomenon, consisting of a series of tumour-host cell interactions. One of these interactions is the infiltration of the cells through vascular endothelium in the organ where secondary tumours will eventually develop (Dingemans, 1973, 1974). In order to study the mechanisms behind this phenomenon in a well-defined environment, we have attempted to use the perfused liver as an experimental model. In these experiments, the tumour cells were added to a defined perfusion medium, consisting of Krebs–Ringer buffer supplemented with glucose and albumin. The livers were fixed at different intervals after addition of the tumour cells, and tumour-liver cell interactions were observed with the electron microscope.

Previously, we have given a detailed description of the infiltration process as observed in lymphosarcoma cells in the perfused liver (Roos *et al.*, 1977). These lymphosarcoma cells were known to infiltrate the liver in large numbers, producing diffuse tumour growth all over the liver. Indeed, in the perfused liver, 10–35% of the lymphosarcoma cells were in an extravascular position within 3 h. Recently, it was shown that leucocytes infiltrated the liver in a similar way (Dingemans *et al.*, 1978). Thus the possibility remained that this way of infiltration was specific for lymphoid cells and might not occur with other tumour cell types. We therefore repeated our experiments with a non-lymphoid tumour, the TA3 ascites mammary carcinoma. This tumour is of particular interest because there are two sublines, TA3/Ha and TA3/St, which differ in cell-surface properties. TA3/Ha cells are covered with a thick cell coat (Miller *et al.*, 1977) consisting of sialoglycoprotein, and consequently have a much higher negative surface charge than TA3/St cells, which lack this coat (Friberg, 1972). As these cell-surface properties might influence the interaction with vascular endothelium, both sublines were studied.

Interactions between macrophages and tumour cells have been studied extensively. However, most observations have been *in vitro* on activated peritoneal macrophages, or macrophages isolated from solid neoplasms. In contrast, little is known about interactions between tumour cells and resident tissue macrophages. The liver contains a large number of resident macrophages, the Kupffer cells. In our studies on lymphosarcoma cells (Dingemans, 1973; Roos *et al.*, 1977) little interaction with Kupffer cells was observed. In the present investigation, however, we have seen apparently intact TA3 cells, particularly TA3/Ha cells, that were being surrounded by Kupffer cell processes. Later, some cells that were completely encircled in the plane of the section were degenerated. We feel that these observations strongly suggest that the TA3/Ha cells were phagocytosed by the Kupffer cells.

In order to demonstrate the validity of the observations in the perfused liver, TA3 cells were also injected *in vivo* into the portal system of syngeneic A mice. Observations in the livers of these mice were compared to those in the perfused liver. Some of these mice were allowed to survive long enough for tumour nodules to develop in the liver.

### MATERIALS AND METHODS

*Tumour cells.*—The TA3 mammary adenocarcinoma originated spontaneously in an A/HeHa mouse in 1949. The solid tumour was converted into ascites tumours on two separate occasions, and the resulting cell lines were maintained in syngeneic A mice by Dr T. S. Hauschka (TA3/Ha) and Dr G. Klein (TA3/St). After several years, the TA3/Ha cell line, without any known pressure, lost its strain specificity and became transplantable in allogeneic mice, and also in rats and hamsters. The TA3/St tumour retained its strain specificity. The history of the 2 cell lines has been described by Hauschka *et al.* (1971).

The cells, which were kindly supplied by Dr G. Klein, were maintained by weekly i.p. passage of $5\times10^4$ TA3/Ha or $5\times10^6$ TA3/St cells in 0·1 ml phosphate-buffered saline (PBS) in syngeneic A mice. The yield after 7 days was $\sim3\times10^8$ TA3/Ha cells in ~2 ml ascitic fluid and $\sim10^8$ TA3/St cells in very little ascitic fluid.

More than 99% of the cells excluded trypan blue. Samples of tumour-cell suspensions were fixed by dilution with an equal volume of 2·5% glutaraldehyde in 0·1M cacodylate buffer, and processed for electron microscopy. For perfusion experiments, the cell suspensions were diluted in PBS and used immediately, or stored at 20°C, but never used later than 30 min after collection. In some experiments the cells were washed twice with PBS, which treatment did not influence the results.

*Mice.*—Livers of both syngeneic A and allogeneic (C57BL×DBA)$F_1$ mice were perfused.

*Perfusion technique.*—The perfusion procedure has been described previously (Roos *et al.*, 1977). Briefly, mouse livers were perfused *in situ* through the vena porta with an oxygenated Krebs–Ringer buffer supplemented with glucose, albumin and amino acids, without an oxygen carrier. Oxygenation and perfusion took place in a thermostatically controlled apparatus at ~35°C. By applying a hydrostatic pressure of 7–8 cm $H_2O$, a flow rate of 3 ml/min was obtained, resulting in an effluent $O_2$ concentration of 2·5–3 parts/$10^6$. We injected $4\times10^6$ TA3/Ha cells or $3\times10^6$ TA3/St cells, which were the largest numbers that could be injected without causing a considerable decrease in flow rate. The cells were injected into the vena porta cannula over a 10-min period.

*Fixation.*—Livers were fixed at intervals ranging from 30–120 min after tumour-cell injection by perfusion with 1·5% glutaraldehyde in 0·067M cacodylate buffer+1% sucrose, postfixed in 1% $OsO_4$ for 2 h (1 $mm^3$ blocks) and processed for electron microscopy. Sections, contrasted with uranyl acetate and lead oxide, were observed in a Philips 301 electron microscope.

*Experiments* in vivo.—Tumour cells were collected in the same way and suspended in the same buffer as in the perfusion experiments, Syngeneic A mice were anaesthetized with ether, and $3 \times 10^6$ cells in 0·2 ml PBS were injected into a small mesenteric vein (Dingemans, 1973, 1974). The livers of 19 mice that had received TA3/Ha or TA3/St cells were fixed by perfusion with glutaraldehyde *via* the portal vein at intervals ranging from 5 min to 10 days after injection.

### RESULTS

### *Morphology of TA3 cells*

Suspensions of both TA3/St and TA3/Ha cells contained single cells and cell clumps of mostly less than 5 cells. Occasionally larger aggregates were present. Cells in clumps were connected by junctions (Fig. 1), indicating that these ascites cells were still epithelial in character. The cells had many intracisternal virus particles which made them easily recognizable in the liver (Fig. 12). TA3/Ha cells were characterized by a cell coat (Fig. 1), which was not observed on TA3/St cells (Fig. 3). The thickness of the coat varied, however, and was not always as prominent as in Fig. 1. The ultrastructure of both cell lines has been described in more detail by Miller *et al.* (1977).

### *Tumour-cell arrest and distribution*

In some experiments effluent perfusion medium was collected, and analysed in an electronic particle counter. We found no significant difference in counts between samples collected before, and up to 2 h after addition of cells. Thus, apparently all tumour cells were arrested in the liver. In the electron microscope we observed that the tumour cells were arrested in the beginning of sinusoids, in the vicinity of portal-vein branches.

### *Interaction with endothelium*

Within 30 min after injection, both TA3/Ha and TA3/St cells extended numerous finger-like protrusions into the sinusoidal endothelium. Some protrusions were seen in invaginations of the endothelial cells, which were generally "bristle-coated" (Fig. 2 and 4), but most protrusions extended through narrow openings in the endothelial cells into the space of Disse (Fig. 3). Even where the distance between the tumour-cell body and the endothelium was considerable, long protrusions often penetrated the endothelium (Fig. 4). Protrusions traversed through openings in both thinner and thicker parts (Fig. 3) of endothelial cells, with no preference for the fenestrated areas of the endothelium.

Of the cells that were seen to penetrate, an average of $3 \pm 2$ protrusions were extended through openings in the endothelium in a section, but as many as 20 protrusions was not exceptional. Since one protrusion can only be seen in a few consecutive sections, and more than 200 sections were needed to completely cut one tumour cell, it is evident that most tumour cells extended hundreds of protrusions through the endothelium.

### *Interaction with hepatocytes*

Part of the extended protrusions not only traversed the endothelium, but also intruded into hepatocytes. Invaginations thus formed in hepatocytes were mostly "bristle-coated" (Fig. 5). TA3/Ha cell protrusions were sometimes observed inside larger invaginations, lacking a "bristle-coat" (Fig. 6). Such protrusions always had a conspicuous cell coat. Of those cells that intruded into hepatocytes, an average of two protrusions per section per cell were seen in hepatocyte invaginations.

Some of the tumour cells that invaginated hepatocytes did so with larger globular cell processes (Fig. 7, 9 and 11). From these processes small protrusions were often extended into hepatocytes (Fig. 9). Occasionally, tumour cells were observed to be situated mostly (Fig. 9) or completely outside the blood vessels (in the plane of the section). Cells were also seen to infiltrate in clusters (Fig. 8).

### *Progress of infiltration with time*

In each experiment we observed at least 100 tumour cells, and determined the per-

FIG. 1.—Part of a TA3/Ha cell clump in a liver sinusoid. The cells are connected by junctions (arrows). Note the thick cell coat. A tumour-cell protrusion extends into another tumour cell (double arrow).

FIG. 2.—A TA3/St cell protrusion (arrow) extends into a "bristle-coated" invagination of an endothelial cell.

FIG. 3.—A TA3/St cell protrusion projects through an opening in a thicker part of an endothelial cell into the space of Disse.

FIG. 4.—Two TA3/Ha protrusions penetrating endothelium. One (arrow) is still in an invagination. A second has just traversed the endothelial cell (double arrow). Note the large distance between tumour cell and endothelium.

FIG. 5.—A TA3/Ha protrusion has traversed a thin part of an endothelial cell and extends into a "bristle-coated" invagination of a hepatocyte.

FIG. 6.—TA3/Ha cell protrusions into a hepatocyte. Note the large invaginations in the hepatocyte and the cell coat on the protrusions.

Symbols: E, endothelial cell; T, tumour cell; H, hepatocyte; bc, bristle-coat.

FIG. 7.—A TA3/Ha cell intruding into hepatocyte with two large globular processes.

FIG. 8.—Two TA3/Ha cells connected by a junction (arrow) are infiltrating liver tissue. One of the cells is outside the vessel (in this section).

FIG. 9.—A TA3/Ha cell situated almost completely outside the blood vessel, intrudes into hepatocytes with large globular processes. A protrusion is extended from a process (arrow). SL, sinusoid lumen.

FIG. 10.—*Infiltration of liver by TA/Ha and TA3/St cells.* In each experiment at least 100 tumour cells were observed in thin sections with the electron microscope. Cells floating in a large vessel (<10%) were not included. Closed symbols: TA3/Ha, open symbols: TA3/St. Circles: Total percentage of cells in any stage of the infiltration process. Triangles: Percentage of cells intruding into hepatocytes. Squares: Percentage of cells intruding into hepatocytes with large cell processes. For a good comparison between the two sublines interacting differently with Kupffer cells, we excluded cells that were more than 50% surrounded by Kupffer cell processes, whether they infiltrated or not.

centage that were in any stage of the infiltration process, the percentage that intruded hepatocytes, and the percentage that did so with large cell processes (Fig. 10). These parameters showed little tendency to increase more than 30 min after injection. The few cells in a (nearly) extravasular position (less than 0·5%) were mostly seen in experiments of longer duration.

*Interaction with Kupffer cells*

Some tumour cells were, in the plane of the section, partly or completely surrounded by sometimes very thin rims of electronlucent and mostly organelle-free cytoplasm (Fig. 11 and 12). In favourable sections (Fig. 11) such rims were seen to be part of liver macrophages (Kupffer cells). Tumour cells in the process of being encircled were morphologically intact and seemed viable and active, as judged from their often infiltrating liver tissue (Fig. 11 and 15). Frequently, Kupffer-cell processes intruded into the tumour cells from opposite directions, in an apparent attempt to occupy part of the tumour cell. Thus tumour cells, and often tumour-cell nuclei also were divided into two parts connected by a thin compressed string of nuclear and cytoplasmic material. Blebs on the nuclear surface suggested that nucleoplasm was actually squeezed out during this process (Fig. 16).

Fig. 14 shows the percentage of tumour cells interacting with Kupffer cells in the plane of the section at different times after addition of the cells to the medium. About 40% of TA3/Ha cells were seen to interact with Kupffer cells. For each of these cells we estimated the percentage of cell surface that was covered by Kupffer cell. The average of these percentages is also shown. One would expect an increase in this average percentage with time, but it did not increase after 30 min. Thus, encirclement did not continue beyond a time when many tumour cells were only partly surrounded.

Interaction of TA3/St cells with Kupffer cells varied considerably, but was generally smaller, often much smaller, than of TA3/Ha cells.

In experiments lasting 1 h or longer, an electron-dense amorphous material was often seen round tumour cells completely surrounded by Kupffer cell (Fig. 13, 17 and 18). This material was probably of lysosomal origin, because electron-dense organelles, presumably lysosomes, were sometimes seen within it (Fig. 17) and also release of this material from lysosomes was occasionally seen (Fig. 18). In experiments lasting 2 h or more, but not earlier, a number of ingested tumour cells was in an advanced stage of degradation (Fig. 13). Often, Kupffer cells contained degraded

FIG. 11.—Two TA3/Ha cells almost completely surrounded by processes of one Kupffer cell (K). One of these cells ($T_1$) is intruding into hepatocytes at 2 sites (arrows).

FIG. 12.—TA3/Ha cell completely surrounded by Kupffer-cell processes. Note the large number of intracisternal virus particles.

FIG. 13.—Kupffer-cell processes surround a degenerating TA3/Ha cell. Electron-dense material is present between Kupffer cell and tumour cell (arrow).

FIG. 14.—*Interaction of TA3 cells with Kupffer cells.* In each experiment at least 100 tumour cells were observed in thin EM sections. Closed symbols: TA3/Ha; open symbols: TA3/St. (a) percentage of cells partly or completely surrounded by Kupffer-cell processes; (b) for each interacting cell, percentage of the surface covered by Kupffer cell, was estimated. The average of these percentages is shown.

cell remnants, usually no longer recognizable as tumour cells. Occasionally, however, intact virus particles were seen in the remnants, indicating their tumour-cell origin.

In the livers of allogeneic (C57BL × DBA) $F_1$ mice, Kupffer-cell interaction with TA3 cells was comparable to that in livers of syngeneic A mice.

*Intrusion of endothelium and hepatocytes by Kupffer cells*

Kupffer cells interacting with tumour cells extended a much larger number of protrusions than usual into and through the endothelium and into hepatocytes. The invaginations surrounding the protrusions were often "bristle-coated" (Fig. 19). Protrusions extending into bristle-coated invaginations were also seen in interactions between leucocytes and liver endothelium (Dingemans, 1978), and also between clustered TA3 tumour cells (Fig. 1).

*Experiments* in vivo

The observations on TA3/Ha and TA3/St cells injected into the portal system of intact mice were remarkably consistent with the observations made in the perfused liver. Virtually all morphological details described above were also observed *in vivo*. Preliminary counts indicated that at 1–6 h after injection, about 40% of the TA3/Ha cells were partially or completely sur-

FIG. 15.—A TA3/Ha cell intruding into a hepatocyte with several processes (arrows) is being encircled by a Kupffer cell. One of the processes appears to be squeezed off the tumour cell (double arrow).
FIG. 16.—A TA3/Ha cell being surrounded by a Kupffer cell. Part of the cell is squeezed off. The nucleus is divided into two parts connected by a thin string of nucleoplasm (arrow). Note the blebs on the nuclear surface (double arrow).
FIG. 17.—Electron-dense material surrounding a TA3/Ha cell inside a Kupffer-cell phagosome. An intact dense body, probably a lysosome, is present within the dense material (arrow).
FIG. 18.—A TA3/Ha cell inside a Kupffer-cell phagosome. Dense material is released into the space between Kupffer cell and tumour cell.
FIG. 19.—A Kupffer cell surrounding a TA3/Ha cell extends several protrusions into an endothelial cell (arrows). Two of these invaginations are bristle-coated.

H
K
T
0.1 µ
15

E
T
K
K
T
E
1 µ
K
16

K
T
17

K
T
18

bc
K
E
T
1 µ
bc
19

rounded by Kupffer cells in the plane of the section, whereas surrounding of TA3/St cells by Kupffer cells was exceptional. These data are in agreement with those obtained in the perfused liver (Fig. 14).

In a few mice that were allowed to survive long enough for large tumour nodules to develop, there was a striking difference between the numbers of such nodules produced by TA3/Ha and TA3/St cells. Whereas $3 \times 10^6$ TA3/Ha cells yielded very few nodules, injection of the same number of TA3/St cells resulted in hundreds of nodules all over the liver.

### DISCUSSION

Our observations seem to offer a nearly complete picture of the infiltration of liver tissue by TA3 mammary carcinoma cells and of their interactions with Kupffer cells. In addition, the remarkably close correspondence between results obtained in perfused livers and *in vivo* strongly suggests that our perfused-liver system is a reliable model for experimental investigation of the mechanisms of these processes.

The following sequence of events can be constructed from our observations on infiltrating TA3 cells. Numerous tumour-cell protrusions touch endothelial cells and induce invaginations that can transform into openings, through which the protrusions project into the space of Disse. These openings are situated in both thinner and thicker parts of endothelium, with no preference for already fenestrated areas. Upon contact of the protrusions with hepatocytes, these cells invaginate too. The protrusions then become globular in shape, bulge and intrude deeply into the hepatocytes. Ultimately, the whole tumour cell may become situated outside the blood vessel. This sequence of events is similar to that described for lymphosarcoma cells (Roos *et al.*, 1977). Many of these steps have also been observed during the infiltration of leucocytes into liver (Dingemans, 1978). The main difference between lymphosarcoma and TA3 carcinoma cells was quantitative. Whereas 10–35% of the lymphosarcoma cells were situated outside the blood vessel within 2–3 h (Roos *et al.*, 1977), this was seldom observed with TA3 cells. This might be due to a cessation of the infiltration process after only 30 min (Fig. 10), which did not occur with lymphosarcoma cells. This cessation was not an artefact of our perfused-liver model, since it also occurred *in vivo*. The extension of protrusions has often been seen as an initial step in infiltration by both tumour cells (Fisher and Fisher, 1961; Babai and Tremblay, 1972; Dingemans, 1973; Fasske *et al.*, 1975; Carr *et al.*, 1976; Chew *et al.*, 1976; Roos *et al.*, 1977) and leucocytes (Dingemans, 1978). The ballooning of these protrusions, which then become globular invasive processes, is apparently also important. This event occurred much more often with lymphosarcoma cells (Roos *et al.*, 1977) than with TA3 carcinoma cells, but with both cell types it was poorly reproducible. Which subtle disparities between different experiments caused this variation, is still obscure.

The invaginations of endothelial cells and hepatocytes often had a "bristle-coat", which resembled that of pinocytotic vesicles. The formation of this coat may be simply a reaction to the intrusion by protrusions. However, it may also indicate that these cells are stimulated by the tumour cells to "endocytize" the protrusions, and thus actively form the invaginations around the protrusions. This stimulus might originate from cell-surface constituents on, or shed from, the tumour cell. We emphasize, however, that the above phenomenon is not specific for tumour cells, as it was observed by us in several cell–cell interactions, *e.g.* between Kupffer cells and endothelium (Fig. 19), between 2 TA3 cells (Fig. 1) and between leucocytes and endothelium (Dingemans, 1978).

TA3/Ha cells have a thick cell coat (Miller *et al.*, 1977), probably consisting of a sialoglycoprotein that is absent on TA3/St cells (Codington *et al.*, 1973). This sialoglycoprotein has been assumed to mask $H2^a$ antigens on TA3/Ha cells (Sanford *et*

*al.*, 1972; Friberg and Liliehöök, 1973), thus rendering them allotransplantable. We noted little difference in the percentage of infiltrating cells between the two cell lines, so the infiltrative capacity of TA3/Ha cells does not seem to be influenced by this cell coat. Because of the large amount of sialic acid on their surface, TA3/Ha cells have a higher surface charge than TA3/St cells (Friberg, 1972). Apparently this does not influence their infiltrative capacity either.

Interactions between tumour cells and macrophages have been much investigated, generally *in vitro*, with activated peritoneal macrophages or macrophages isolated from solid neoplasms. There is general agreement that *in vitro* destruction of tumour cells by non-immune macrophages is a non-phagocytic process (Alexander and Evans, 1971; Hibbs, 1976; Keller 1976). *In vivo*, both non-phagocytic (Snodgrass and Hanna, 1973) and phagocytic Carr *et al.*, 1974) mechanisms have been reported.

In our previous studies on lymphosarcoma cells, some tumour cells were seen to be tightly bound to Kupffer cells (Dingemans, 1973), but destruction by Kupffer cells was not seen. In the present study, however, we have frequently seen the encirclement of TA3 cells, particularly TA3/Ha cells, by Kupffer-cell processes. Tumour cells being encircled by Kupffer cells were not just dead or damaged cells, since they were frequently seen to be actively infiltrating. Initially, encircled cells were morphologically intact. Only in experiments of longer duration were damaged tumour cells seen completely surrounded by Kupffer cell in the plane of the section. This degeneration was probably due to lysosomal enzymes released by the Kupffer cell (Fig. 17 and 18).

We feel that these observations strongly suggest that Kupffer cells phagocytosed, or attempted to phagocytose, intact TA3/Ha cells. Similar observations on both non-specific and immunologically specific macrophage-mediated tumour-cell destruction have likewise been referred to as "apparent phagocytosis" (Carr *et al.*, 1974; Chambers and Weiser, 1972, 1973). Many details described by Chambers and Weiser, in particular, were strikingly similar to our own observations.

Interaction with Kupffer cells occurred without prior sensitization of the animals to the tumour cells and to the same extent in livers of syngeneic and allogeneic mice. In the perfused liver, encirclement of tumour cells occurred in the absence of serum opsonins. *In vivo*, where serum opsonins were probably present, interaction did not occur more often. Thus, for this tumour serum opsonins do not seem to be essential for macrophage-mediated destruction, as has been suggested for other tumours (DiLuzio *et al.*, 1974).

Kupffer cells released an amorphous material round completely encircled tumour cells. This has also been observed by Chambers and Weiser (1973), who assumed that it was lysosomal material. In addition, we have seen in this material dense organelles, presumably intact lysosomes. This release of lysosomes has also been reported for non-phagocytic tumour-cell destruction (Hibbs, 1974; Bucana *et al.*, 1976).

In preliminary experiments, B16 melanoma cells were also frequently seen to be encircled by Kupffer cells (Roos *et al.*, unpublished), indicating that the phenomenon is not restricted to the unusual TA3/Ha cell.

The establishment of a limited number of nodular metastases by TA3 cells *in vivo*, as compared to the diffuse growth pattern of lymphosarcoma cells (Dingemans, 1973), might be explained by the relatively low percentage of cells that succeeded in reaching an extra-vascular position, namely less than 0·5%, compared with 10–35% of lymphosarcoma cells (Roos *et al.*, 1977). The striking difference in number of tumour nodules which developed from equal numbers of TA3/Ha and TA3/St cells respectively is possibly due to the higher susceptibility of TA3/Ha cells to phagocytosis by Kupffer cells. Work is in progress to evaluate this possibility.

We are indebted to Dr G. Klein, Karolinska Institute, Stockholm, Sweden for providing the TA3 cells. We thank Miss C. Koning and Mr N. Ong for preparing micrographs.

### REFERENCES

ALEXANDER, P. & EVANS, R. (1971) Endotoxin and double-stranded RNA render macrophages cytotoxic. *Nature, New Biol.*, **232**, 76.

BABAI, F. & TREMBLAY, G. (1972) Ultrastructural study of liver invasion by Novikoff hepatoma. *Cancer Res.*, **32**, 2765.

BUCANA, C., HOYER, L. C., HOBBS, B., BREESMAN, S., MCDANIEL, M. & HANNA, M. G. (1976) Morphological evidence for the translocation of lysosomal organelles from cytotoxic macrophages into the cytoplasm of tumor target cells. *Cancer Res.*, **36**, 4444.

CARR, I., UNDERWOOD, J. C. E., MCGINTY, F. & WOOD, P. (1974) The ultrastructure of the local lymphoreticular response to an experimental neoplasm. *J. Pathol.*, **113**, 175.

CARR, I., MCGINTY, F. & NORRIS, P. (1976) The fine structure of neoplastic invasion: invasion of liver, skeletal muscle and lymphatic vessels by the Rd/3 tumor. *J. Pathol.*, **118**, 91.

CHEW, E. C., JOSEPHSON, R. L. & WALLACE, A. C. (1976) Morphologic aspects of the arrest of circulating cancer cells. In *Fundamental Aspects of Metastasis*, Ed. L. Weiss Amsterdam: North Holland. p. 121.

CHAMBERS, V. C. & WEISER, R. C. (1972) The ultrastructure of Sarcoma I cells and immune macrophages during their interaction in the peritoneal cavities of immune C57BL/6 mice. *Cancer Res.*, **32**, 413.

CHAMBERS, V. C. & WEISER, R. C. (1973) An electron microscope study of the cytophagocytosis of Sarcoma I cells by alloimmune macrophages. *J. Natl. Cancer Inst.*, **51**, 1369.

CODINGTON, J. F., SANFORD, B. H. & JEANLOZ, R. W. (1973) Cell-surface glycoproteins of two sublines of the TA3 tumor. *J. Natl. Cancer Inst.*, **51**, 595.

DILUZIO, N. R., MCNAMEE, R., OLCAY, I., KITAHAMA, A. & MILLER, A. H. (1974) Inhibition of tumor growth by recognition factors. *Proc. Soc. Exp. Biol. Med.*, **145**, 311.

DINGEMANS, K. P. (1973) Behaviour of intravenously injected malignant lymphoma cells. A morphologic study. *J. Natl. Cancer Inst.*, **51**, 1883.

DINGEMANS, K. P. (1974) Invasion of liver tissue by blood-borne mammary carcinoma cells. *J. Natl. Cancer Inst.*, **53**, 1813.

DINGEMANS, K. P., ROOS, E. VAN DEN BERGH-WEERMAN, M. A. & VAN DE PAVERT, I. V. (1978) Invasion of liver tissue by tumor cells and leucocytes. Comparative ultrastructure. *J. Natl. Cancer Inst.* **60**, 583.

FASSKE, E., FETTING, R., RUHLAND, D., SCHUBERT, T. & THEMANN, H. (1975) Colonization of the mouse liver by transplanted virogenic leukemia cells. Electron microscopic investigation. *Z. Krebsforsch.*, **84**, 257.

FISHER, E. R. & FISHER, B. (1961) Electron microscopic, histologic, and histochemical features of the Walker carcinoma. *Cancer Res.*, **21**, 527.

FRIBERG, S. (1972) Comparison of an immunoresistant and immuno-susceptible ascites subline from murine tumor TA3. I. Transplantability, morphology and some physico-chemical characteristics. *J. Natl. Cancer Inst.*, **48**, 1463.

FRIBERG, S. & LILIEHÖÖK, B. (1973) Evidence for non-exposed H2 antigens in immunoresistant murine tumor. *Nature, New Biol.*, **241**, 112.

HAUSCHKA, T. S., WEISS, L. HOLDRIDGE, B. A., CUDNEY, T. L., ZUMPFT, M. & PLANINSEK, J. A. (1971) Karyotypic and surface features of murine TA3 carcinoma cells during immunoselection in mice and rats. *J. Natl. Cancer Inst.*, **47**, 343.

HIBBS, J. B. (1974) Heterocytolysis by macrophages activated by Bacillus-Calmette–Guerin: lysosome exocytosis into tumor cells. *Science*, **184**, 468.

HIBBS, J. B. (1976) The macrophage as a tumoricidal effector cell: a review of *in vivo* and *in vitro* studies on the mechanism of the activated macrophage non-specific cytotoxic reaction. In *The Macrophage in Neoplasia*, Ed.: M. A. Fink. New York: Acad. Press. p. 83.

KELLER, R. (1976) Cytostatic and cytocidal effects of activated non-immune macrophages. In *The Macrophage in Neoplasia*, Ed. M. A. Fink, New York: Academic Press. p. 149.

MILLER, S. C., HAY, E. D. & CODINGTON, J. F. (1977) Ultrastructural and histochemical differences in cell surface properties of strain-specific and non-strain-specific TA3 adenocarcinoma cells. *J. Cell Biol.*, **72**, 511.

ROOS, E., DINGEMANS, K. P., VAN DE PAVERT, I. V. & VAN DEN BERGH-WEERMAN, M. A. (1977) Invasion of lymphosarcoma cells into the perfused mouse liver. *J. Natl. Cancer Inst.*, **58**, 399.

SANFORD, B. H., CODINGTON, J. F. JEANLOZ, R. W. & PALMER, P. D. (1972) Transplantability and antigenicity of two sublines of the TA3 tumor. *J. Immunol.*, **110**, 1233.

SNODGRASS, M. J. & HANNA, M. G. (1973) Ultrastructural studies of histiocyte-tumor cell interactions during tumor regression after intralesional injection of *Mycobacterium bovis*. *Cancer Res.*, **33**, 701.